# 616 Application of Antibacterial Thermosensitive Collagen-Based Hydrogel in Wound Healing

**DOI:** 10.1093/jbcr/irac012.244

**Published:** 2022-03-23

**Authors:** Nafise Amiri, Sahand Ghaffari, Ida Hassanpour, Taesik Chae, Reza B Jalili, Ruhangiz Taghi Kilani, Frank Ko, Dirk Lange

**Affiliations:** University of British Columbia, Burn and Wound Research Group, Vancouver, British Columbia; University of British Columbia, Vancouver, British Columbia; Burn and Wound Healing Research Lab, The University of British Columbia, Vancouver, British Columbia; University of British Columbia, Vancouver, British Columbia; University of British Columbia, Vancouver, British Columbia; UBC, Vancouver, British Columbia; University of British Columbia, Vencouver, British Columbia; University of British Columbia, Vancouver, British Columbia; UBC, Vancouver, British Columbia

## Abstract

**Introduction:**

Burn wound infections are a serious complication of thermal injury. Among the many factors that may limit effective wound healing in patients with burn, bacterial infection and poor cell recruitment appear as the leading causes for prolonged healing. Thus, a novel strategy that aims to prevent bacterial infection within the wound, while at the same time providing structural scaffolding that promotes endogenous tissue repair, would be of great interest. As a nutritional protective barrier for the wound, we developed a thermosensitive collagen-based matrix called MeshFill (MF) that contains all nutrition required for cell growth with the ability to fill up all the cavities and void areas in wounds regardless of their geometry. In a previous study, MF was successfully combined with partial-thickness mesh grafted skin in a porcine model and improved healing and aesthetic outcomes. In the present work, we report on the development, and in vitro and in vivo testing of a new formulation of MF containing silver nanoparticles (AgNPs), which simultaneously prevent bacterial infection and promote skin regeneration.

**Methods:**

We fabricated MF/Ag formulation by loading different concentrations of AgNPs in MF hydrogel. The antibacterial activity of MF/Ag formulation against *Methicillin-resistant Staphylococcus aureus* (MRSA) and *Pseudomonas aeruginosa* (PA) was examined in vitro. The wound healing efficacy of the formulation was evaluated in a silicon ring splinted delayed wound healing model in rats. The splinted full-thickness wounds were generated on the back of rats and treated with either MF or MF/Ag with different concentrations of AgNPs or were bandaged with no treatment (NT) as a control. The healing process was monitored for 18 days. Clinical wound measurements and histological assessments were performed to compare different treatment regimens

**Results:**

The results of in vitro antibacterial study showed MF/Ag released a sufficient concentration of silver which caused a marked reduction in colony forming units (CFU) of MRSA and PA as compared to MF alone. MF/Ag did not show any cytotoxicity to human fibroblast. Moreover, the result of the animal study confirmed the safety and efficacy of applying different concentrations of AgNPs loaded in MF without compromising the healing outcome in our rat model.

**Conclusions:**

These findings suggest that AgNPs loaded MF would be a safe, nutritional, flowable hydrogel that provides an ideal moisture environment for healing while protecting the wound from bacteria and can potentially be used as a functional scaffold in partial-thickness mesh grafted skin in burn patients.

